# In-depth analysis of the tear fluid glycoproteome reveals diverse lacritin glycosylation and spliceoforms

**DOI:** 10.1016/j.jbc.2025.110580

**Published:** 2025-08-08

**Authors:** Vincent Chang, Keira E. Mahoney, Isaac Lian, Ryan Chen, Nara Chung, Tor Paaske Utheim, Niclas G. Karlsson, Stacy A. Malaker

**Affiliations:** 1 Department of Chemistry, Yale University, New Haven, Connecticut, USA; 2 Department of Medical Biochemistry, Oslo University Hospital, Oslo, Norway; 3 Department of Life Sciences and Health, Faculty of Health Sciences, Oslo Metropolitan University Oslo Metropolitan University, Oslo, Norway

**Keywords:** glycoproteomics, lacritin, immunoglobulin A, tear fluid, mass spectrometry, mucin, mucinase

## Abstract

Tear fluid comprises a diverse group of extracellular glycoproteins which are critical for ocular homeostasis. Within the tear fluid glycoproteome, lacritin is highly expressed and plays a key role in immune response, tear secretion, and antimicrobial activity. Importantly, glycosylation constitutes over 50% of lacritin’s molecular weight. However, despite this fact, nothing is known about the specific glycan structures on lacritin and how they influence its protein folding, function, or downstream biological processes. Similarly, it remains completely unknown whether alterations to lacritin glycans are correlated with ocular pathologies. To address this gap in knowledge, we harnessed mass spectrometry to conduct the first O-glycoproteomic study of tear fluid. Here, we report unprecedented coverage of lacritin glycosylation, detailing 19 O-glycosites bearing a myriad of glycan structures. Further, we leveraged AlphaFold 3.0 and GlycoShape to visualize the impact of these glycans on its structure, demonstrating that O-glycosylation renders the protein backbone rigid and extended. Surprisingly, we also detected protein-level evidence of two lacritin spliceoforms, representing the first observation of these isoforms by mass spectrometry. Simultaneously, we describe the most comprehensive characterization of the tear fluid glycoproteome to date, elucidating the glycosylation profile of immunoglobulin A, lactoferrin, and other glycoproteins with demonstrated clinical relevance as diagnostic biomarkers. Overall, this study lays the critical groundwork for future biochemical investigation of tear fluid glycoproteins and their application as diagnostic or therapeutic tools for ocular diseases.

Tears are a complex extracellular fluid comprised of lipids, electrolytes, small-molecule metabolites, and glycoproteins (1). Together, these biomolecules play key roles in maintaining ocular homeostasis and epithelial health. In dry eye disease (DED), tissue integrity and corneal maintenance is disrupted, resulting in chronic pain, impaired vision, and lasting corneal dysfunction (2, 3, 4). Currently, DED affects 5% to 50% of the world’s population (5, 6), yet limited molecular diagnostics exist while therapies only provide modest relief to patient symptoms. Within the tear fluid glycoproteome, lacritin stands out as a glycoprotein essential for tear secretion, eye lubrication, antimicrobial activity, and immune modulation (7, 8). Given its critical role at the ocular surface, it is perhaps unsurprising that lacritin expression levels change significantly in DED (9, 10, 11); as such, it is currently being investigated as a diagnostic biomarker. Beyond its diagnostic capacity, lacritin bears a peptide epitope (residues 114–138) that has recently been developed into Lacripep, a first-in-class therapeutic candidate for DED which aims to reverse pathological outcomes of dry eye (12, 13).

These advances in lacritin-based diagnostics and therapeutics are largely driven by key biochemical discoveries made by the Laurie group over the past 3 decades. Previously, they demonstrated that the biological activity of lacritin is mediated by its ability to bind syndecan-1, which initiates calcium signaling and mitogenic activity toward epithelial repair (14, 15). Importantly, signaling through the lacritin–syndecan-1 axis is established through noncovalent interactions between the C-terminal alpha helix of lacritin (residues 114–138) and the N-terminal residues (1–51) of syndecan-1. Interestingly, lacritin is also known to form dimers, trimers, and multimers through transglutaminase 2 (TGM2)-mediated crosslinking, where Lys101 and Lys104 act as donor residues and Gln125 acts as an acceptor residue (16). Although the role of lacritin multimers is unclear, they bind syndecan-1 less effectively than monomeric lacritin, suggesting that multimerization impairs syndecan-1 binding and thus downstream lacritin effector function. In addition, elevated levels of TGM2 are implicated in ocular diseases, such as DED (17) and glaucoma (18), where negative regulation of monomeric lacritin by TGM2 could be an underlying mechanism.

Despite over 30 years of literature on lacritin, the glycan structures that decorate its protein backbone remain completely unknown. Glycans comprise more than half of its molecular weight (19); however, their impact on lacritin structure, function, and protein–protein interactions (PPIs) remains entirely unexplored. Most commonly, glycans attach to asparagine (N-linked) and serine/threonine residues (O-linked) in a nontemplated fashion. Crucially, glycans mediate a host of biochemical processes, including protein folding, cellular adhesion, and immune signaling (20, 21). In addition, dysregulation of glycosylation is concomitant with a wide range of diseases, including ocular pathologies, such as diabetic retinopathy (22) and atopic keratoconjunctivitis/vernal keratoconjunctivitis (23). Overall, molecular-level insight into the glycosylation landscape of lacritin could have significant implications for unraveling biology dictated by its glycans and its potential dysregulation in various diseases. Furthermore, site-specific elucidation of lacritin glycoepitopes could aid in the development of glycosylation-centric diagnostic tools and therapeutic modalities for ocular diseases where lacritin expression and glycosylation are dysregulated.

Glycoproteomics, or the systems-level study of glycoproteins using mass spectrometry (MS), has emerged as the premier analytical strategy to reveal key glycosylation signatures in complex biological samples (20, 21). As lacritin is predicted to be heavily O-glycosylated, we reasoned that a targeted O-glycoproteomic analysis of tear fluid could elucidate its endogenous glycosylation landscape. Currently, however, glycoproteomic profiling of tear fluid presents an enormous analytical challenge because of the low concentration of proteins in tears, low volume of sample collection, and sheer complexity of the glycoproteome. This is further highlighted by the fact that the two existing N-glycoproteomic studies required very large sample input, thus necessitating patient samples to be pooled for analysis (24, 25). In addition, these studies employed the endoglycosidase PNGaseF for analysis. This enzyme removes endogenously expressed N-glycans, leaving a deaminated scar in its place, and is often used to site-localize where N-glycans were present. That said, this technique cannot reveal which glycans are found at individual residues, thus leaving critical information unstudied. Finally, O-glycoproteomic studies have never been performed using tear fluid, further highlighting the difficulty in studying this type of sample.

To address this gap in knowledge, we sought to implement intact glycoproteomics workflows capable of characterizing tear fluid glycoproteins from single patients. Previously, our laboratory developed two O-glycoprotein enrichment strategies that employ a class of recently introduced enzymes termed “mucinases.” Briefly, mucinases are a subclass of proteases that specialize in digestion of proteins bearing densely O-glycosylated domains (herein referred to as mucin domains) to generate glycopeptides amenable to MS analysis (26, 27, 28). In prior studies, we reported the use of two mucinases, *Serratia marcescens* Enhancin (SmE) (28) and secreted protease of C1 esterase inhibitor (StcE) (29), to aid in O-glycoproteomic analysis. In our first enrichment method, an O-glycosylation-focused filter-aided sample preparation (GlycoFASP) workflow generates abundant O-glycopeptides that can be separated from nonglycosylated proteins using a molecular weight cutoff filter (30). The other preparation method employs an inactive point mutant of StcE (StcE^E447D^), which binds mucins without cleaving them. Here, StcE^E447D^ is immobilized onto a solid support to selectively enrich mucin-domain glycoproteins from complex biological samples (31).

Leveraging these methods, here we describe unprecedented coverage of the tear fluid glycoproteome, highlighting diverse lacritin glycoforms bearing highly heterogeneous glycan structures. Building on this, we performed protein modeling with AlphaFold 3.0 and GlycoShape to visualize the chemical influence imparted by glycans on lacritin’s secondary structure. Furthermore, we report protein-level evidence of two lacritin spliceoforms, which may help to explain the diversity of lacritin glycoforms. Finally, beyond lacritin, we elucidate the glycosylation profile of IgA and other glycoproteins known to play key roles at the ocular surface. Overall, the findings from this study lay the critical groundwork for future investigation of lacritin and other tear fluid glycoproteins in ocular diseases such as DED.

## Results and discussion

### Development of a new glycoproteomic workflow for tear fluid

**Figure 1:**
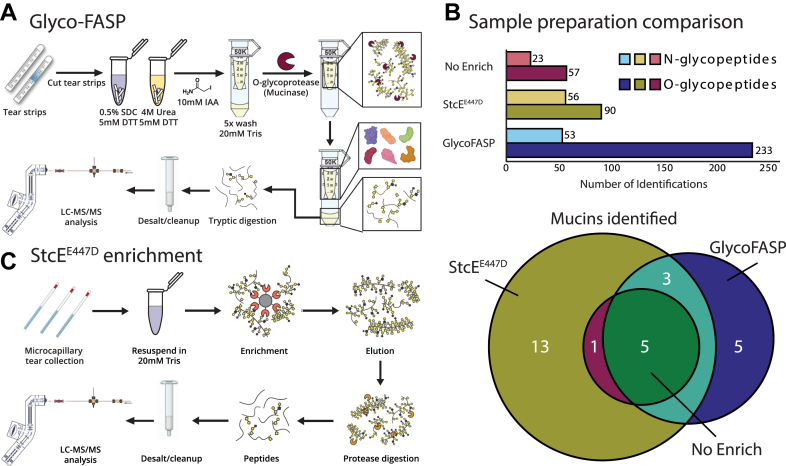
**Glycoproteomic enrichment methods for tear fluid.***A*, GlycoFASP strategy performed on tear fluid proteins extracted from Schirmer tear strips. Proteins are reduced and alkylated following extraction and loaded onto a 50 KDa MWCO filter before protease digestion and subsequent MS analysis. *B*, StcE^E447D^, an inactive mucinase, is conjugated onto a solid support and used to enrich mucin-domain O-glycoproteins from tears collected by capillaries. Following enrichment and elution, proteins are digested with proteases before MS analysis. *C*, sample preparation comparison for N- and O-glycopeptide IDs (*top*) and mucin IDs (*bottom*). In total, 22 mucins were IDed with StcE^E447D^ enrichment, 6 mucins with no enrichment, and 13 mucins with GlycoFASP. GlycoFASP, O-glycosylation-focused filter-aided sample preparation; ID, identification; MS, mass spectrometry; MWCO, molecular weight cutoff.

Here, we adapted our GlycoFASP strategy (30) to analyze tear fluid O-glycoproteins derived from Schrimer tear strips (Fig. 1*A*), where a typical collection yields up to 80 μg of protein (32). Briefly, our method employed a protein extraction protocol where reduction with DTT is performed directly on the Schirmer strip in the presence of a chaotropic agent (Fig. 1*A*). Following extraction, proteins were alkylated with iodoacetamide (IAA) and loaded onto a 50 kDa filter for several rounds of washes. Finally, an O-glycoprotease (mucinase SmE) was added for on-filter protein digestion, and glycopeptides passed through the filter were collected for desalting and subsequent LC–MS/MS analysis. We note that we opted to use SmE as opposed to other O-glycoproteases in this study as we have previously shown that SmE outperformed OgpA and ImpA in generating glycopeptides from mucinous glycoproteins, which comprise a significant portion of the tear fluid glycoproteome (28).

When compared with an unenriched control (representative of proteomic sample processing methods currently reported in the literature), GlycoFASP boasted an ∼4 fold increase in O-glycopeptide (57 to 233) identifications (IDs), and an ∼2 fold increase in N-glycopeptide IDs (23 to 53) (Fig. 1*B* andTable S1). While GlycoFASP enabled us to generate a high abundance of O-glycopeptides, the number of mucin-domain glycoprotein IDs (13 in total) was fewer than we initially anticipated given that abundant mucin expression is essential for ocular homeostasis (33). To improve global ID of mucin-domain O-glycoproteins, we opted for an alternative enrichment workflow, which immobilizes StcE^E447D^ onto a solid support, thus allowing us to selectively enrich mucin-domain glycoproteins from complex samples (Fig. 1*C*) (31). As previously reported, this method requires higher sample input relative to GlycoFASP (300 μg *versus* 50 μg of protein). We therefore reasoned that tears derived from microcapillary sampling, where collections from the same patient throughout the day could yield sufficient protein quantities, would be suitable for this enrichment method. Compared with an unenriched control, StcE^E447D^ enrichment similarly resulted in higher O-glycopeptide IDs (57 to 90) and N-glycopeptide IDs (23 to 56). Notably, however, mucin glycoprotein IDs more than tripled (6 to 22) relative to an unenriched control (Fig. 1*B* and Table S1). This resulted in the identification of MUC5AC, MUC1, and many other mucin-domain glycoproteins which were not detected in the unenriched control. While MUC16 was not identified in this study, it is known to exist in tear fluid at relatively low levels by Western blot (22). We expect that increased tear fluid sample input might enable targeted glycoproteomic characterization of the canonical mucins in future studies. Nonetheless, taken together, these results highlight the versatility of our workflows in capturing the glycosylation landscape of numerous tear fluid glycoproteins without the need to pool tear fluid samples or the application of PNGaseF as in previous glycoproteomic studies. Ultimately, the tools introduced here have significantly expanded on the level of sensitivity and glycoproteomic depth necessary for detailed elucidation of tear fluid glycoepitopes. As we demonstrated that this method can be applied to just a few microliters of tear fluid from a single patient, we envision that our strategies could be adapted to larger cohorts for future investigation of lacritin and other disease biomarkers in ocular pathologies such as DED.

### Glycosylation landscape of lacritin

**Figure 2:**
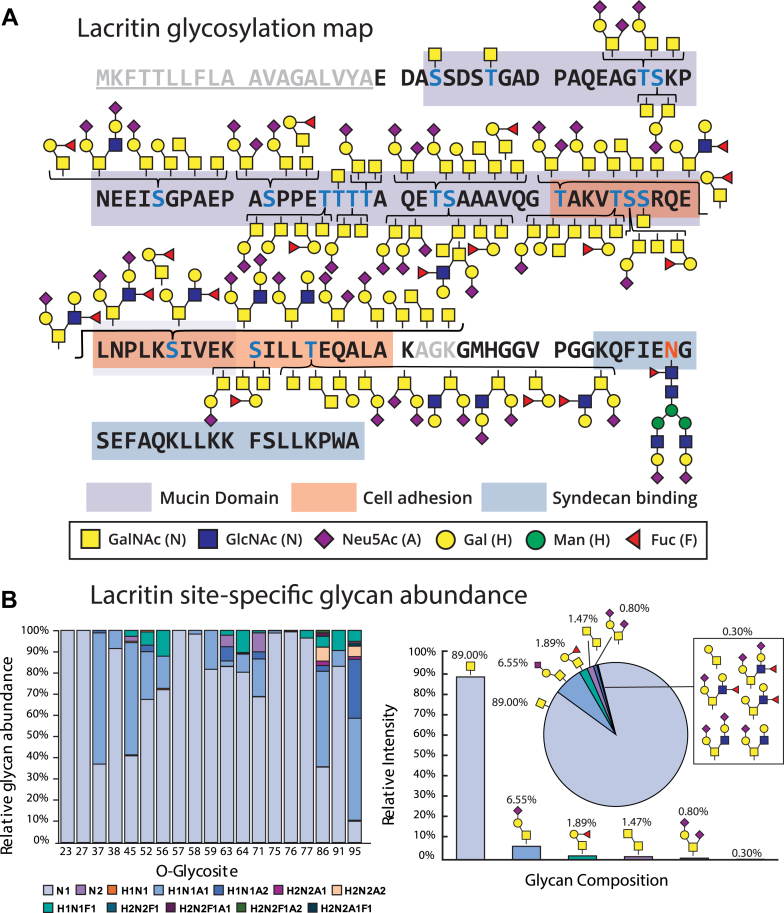
**Glycosylation landscape of lacritin.***A*, full sequence of the canonical isoform of lacritin. The signal sequence is *underlined* and shaded in *gray*, localized O-glycosites are colored in *blue*, and the N-glycosite is colored in *orange*. Regions important for cell adhesion (*orange*), syndecan binding (*blue*), and representing the mucin domain (*purple*) are also shown. *B*, site-specific quantification of lacritin at each glycosite (*left*). Relative O-glycan abundances across all O-glycosites. Quantification was performed using label-free quantitation (LFQ) and area under the curve (AUC) values from extracted ion chromatograms (XICs) of all identified glycopeptides.

Elucidating the site-specific glycan structures that decorate lacritin has significant implications for furthering our molecular-level understanding of lacritin’s biological roles. With this in mind, we harnessed our glycoproteomic workflow to comprehensively map the glycosylation landscape of endogenous lacritin (Fig. 2*A*). Prior to this study, 14 O-glycosites were predicted based on its protein sequence using NetOGlyc 4.0 (19), which leverages a neural network to predict sites of O-linked glycosylation based on experimental evidence. Notably, we detected all 14 predicted sites as well as an additional five O-glycosites that were not predicted by NetOGlyc. Many of these were modified by a range of different glycan structures. For instance, we observed the Tn antigen (GalNAcα1-Ser/Thr; 89% relative abundance), sialylated core 1 (7.35%), type 3 H-antigen (Fuc α1-2-Galβ1-3GalNAcα1-Ser/Thr; 1.89%), and sialylated and fucosylated core 2 (0.30%) O-glycan structures, which were quantified by label-free quantitation (LFQ) using area under the curve (AUC) intensities of extracted ion chromatograms (XICs) (Fig. 2*B*). Overall, the O-glycans we site-localized onto lacritin corroborate many of the O-glycan structures observed in previous glycomic studies of basal tears (34, 35), including the sialyl Lewis x epitope and the H blood group antigen. In addition, we noticed that regions of lacritin known to be important for cell adhesion (36, 37) and syndecan binding (14, 15, 38) contained glycosylated Ser, Thr, or Asn residues. As such, it is likely that distinct lacritin glycoforms have unique roles in tear fluid and future studies to elucidate their specific functions are warranted. Finally, we note that the glycosylation profile of lacritin we obtained is representative of a single patient donor. Therefore, the relative abundances of the elucidated glycan structures may exhibit slight patient-specific variability. Nonetheless, previous studies that employ glycomic profiling (22, 39) and lectin staining (40) of tear fluid indicated relatively low variability across patients. Overall, future exploration of lacritin glycoepitopes for diagnostic purposes will benefit from a substantially larger patient cohort.

### Lacritin protein modeling with GlycoShape and AlphaFold 3.0

**Figure 3:**
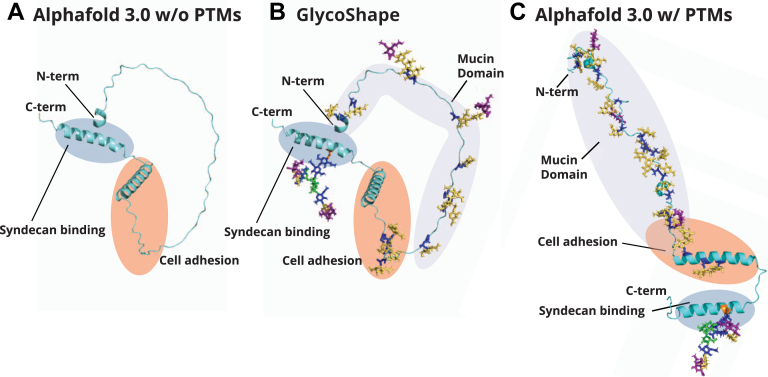
**Protein modeling of lacritin.***A*, current predicted structure of lacritin with AlphaFold 3.0 and no PTMs. *B*, GlycoShape predicted structure of lacritin. *C*, AlphaFold 3.0 structure with PTMs. O-glycans used for GlycoShape and AlphaFold 3.0 are based on only the most abundant O-glycan structure at each glycosite. Regions important for cell adhesion (*orange*), syndecan binding (*blue*), and representing the mucin domain (*purple*) are also shown. Protein backbones are colored in *cyan*, and glycans are colored according to their corresponding SNFG codes. The signal sequence (amino acids 1–19) of lacritin was removed when generating these structures. PTM, post-translational modification; SNFG, Symbol Nomenclature for Glycans.

It is possible that lacritin glycans mediate its PPIs given their proximity to key crosslinking amino acid residues and their ability to dramatically affect protein structure and folding. To address the influence of glycans on the secondary structure of lacritin, we sought to convert our 2D lacritin glycomap into a glycosylated 3D protein structure. To do so, we first calculated the most abundant glycan structure at each glycosite (Fig. 2*B*). Interestingly, the predominant glycan structure was the Tn antigen for all glycosites except Thr37, Ser 45, Ser 86, and Thr95. Next, we leveraged a recent glycoprotein modeling software, GlycoShape (41), which can site-specifically incorporate glycans onto the protein backbone. Importantly, GlycoShape encompasses a wide repertoire of available glycan structures, which made it an attractive starting point for glycan visualization. To date, the best structure prediction of lacritin originates from AlphaFold 3.0 (Fig. 3*A*). Comparatively, the GlycoShape-predicted structure (Fig. 3*B*) highlights the large steric contribution imparted by lacritin N- and O-glycans. While this was a promising start to revise the proposed structure of lacritin, we realized that this model did not significantly change the predicted fold of the protein. Indeed, GlycoShape was primarily designed for visualization of N-glycosite occupancy where the fold of the protein is already structured. For mucin domains that are predicted to be intrinsically disordered, GlycoShape can sample 3D space accessibility at O-glycosites to evaluate steric clashes; however, the base protein fold predicted by AlphaFold remains unchanged. Given the density of O-glycosylation on the lacritin protein backbone, we expected that it might adopt the typical “bottle brush–like” structure characteristic of mucin-domain glycoproteins (28, 42, 43). Inspired by the new post-translational modification feature of AlphaFold 3.0, we repeated the process of adding N- and O-glycans to the backbone of lactrin and, as seen in Figure 3*C*, we observed a dramatic change in the predicted protein fold (or lack thereof, with respect to the mucin domain). Overall, this supports that lacritin glycosylation is predicted to significantly change the secondary structure of lacritin.

In both of the protein models we investigated, glycans were found within regions of lacritin that are important for PPIs. Prior studies by the Laurie group on the lacritin–syndecan-1 axis revealed that the nonglycosylated C-terminal helix of lacritin was sufficient to bind syndecan-1 and induce downstream signaling (14, 15, 44). Given that we observed an occupied N-glycosite within this helix, we first wanted to visualize the steric contribution of this glycan with GlycoShape (Fig. S1). As expected, the size of the glycan within this helix is significant and would likely affect or prevent syndecan-1 binding. We therefore aimed to quantify the relative abundance of this glycoform in comparison to its nonglycosylated counterpart (Fig. S2). Interestingly, the relative abundance of the unmodified peptide was significantly higher, accounting for 99.3% of the relative intensity, whereas the N-glycosylated peptide accounted for only 0.7%. Based on the low abundance and large steric contribution of this N-glycan, it is possible that monomeric populations of lacritin, which engage syndecan-1, do not bear this glycoepitope. The presence of this N-glycan was previously unreported, and its role therefore remains unclear. It may function to affect lacritin C-terminal proteolytic processing, transglutaminase-mediated lacritin multimerization *via* Gln125, or engage another protein entirely. Future biochemical studies with an N-glycosylated helix of lacritin will be necessary to determine the extent to which the N-glycan mediates or abrogates binding to syndecan-1.

## Identification of lacritin isoforms A, C, and D

Based on the vast diversity of lacritin glycoforms we observed, we asked whether splice variants were also detectable and potentially contributing to the heterogeneous population of lacritin. Indeed, several prior studies have demonstrated that protein splice variants could harbor distinct glycosylation signatures (45, 46, 47). Most recently we reported that lubricin, a mucin-domain glycoprotein important for joint lubrication, exhibited multiple splice variants that were modified by isoform-specific O-glycan profiles (48). Here, we reasoned that lacritin splice variants may also be present in our analysis. To date, outside the canonical isoform A, three additional mRNA splice variants of lacritin (isoforms B, C, and D) have been identified by transcriptomics albeit in relatively low abundance (10, 19). Of these isoforms, only isoform C has been detected at the protein level with an isoform-specific antibody (49), though it remains to be validated by MS-based proteomics. Interestingly, isoform D is not predicted to be translated based on its short sequence length and lack of a signal peptide, though the entirety of its O-glycosylated mucin domain is encoded.

**Figure 4:**
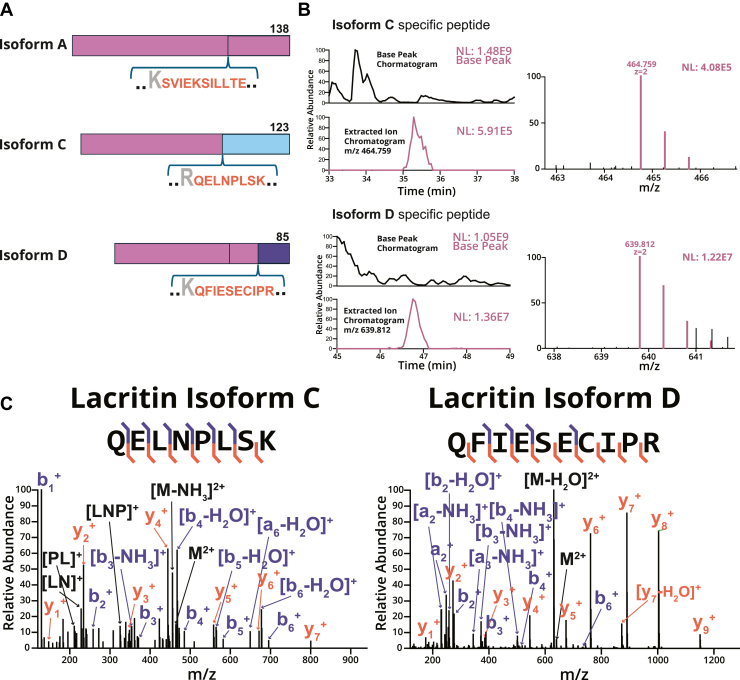
**Lacritin isoforms from alternative splicing.***A*, visual representation of lacritin isoform and sequence-specific peptides. *B*, XIC and base peak chromatogram for the isoform C-specific peptide (*m/z* = 464.759, *z* = 2, retention time = 33–38 min) and the isoform D–specific peptide (*m/z* 639.812, *z* = 2, retention time = 45–49 min). The MS1 of the monoisotopic precursor and its isotopes are shown (*right*) along with other coisolated species. *C*, annotated MS2 spectra for isoform-specific peptides C and D. MS, mass spectrometry; XIC, extracted ion chromatogram.

To investigate the presence of different lacritin splice variants, we first determined tryptic peptide sequences unique to each isoform (Fig. 4*A*). The peptides SILLTEQALAK, QELNPLSK, and QFIESECIPR provided evidence for spliceoforms A, C, and D, respectively. Notably, the tryptic peptide QELNPLKQALAK (containing one missed cleavage) from isoform B is distinct from A and C, though this sequence is shared with isoform D. Regardless, this peptide was not observed in this study, so we cannot comment on the presence of isoform B. After determining these sequences, we extracted the XICs for the peptide QLENPLSK (isoform C) at *m/z* 469.759 and QFIESECIPR (isoform D) at *m/z* 639.812; we then confirmed the sequence assignment using MS2 (Fig. 4*B* and *C*). For isoform A, we identified both glycosylated and unmodified versions of SILLTEQALAK, which was quantified using LFQ of AUC intensities from XICs of each glycoform (Fig. S3). Quantification revealed that the unmodified isoform A peptide had significantly higher relative abundance (6.97∗10^10^ AUC intensity) compared with the three other glycoforms, which exhibited either a Tn antigen or a disialylated core 1 O-glycan (Fig. S3). Furthermore, the intensity of the isoform A-specific peptide (6.97∗10^10^) was much higher compared to that of isoforms C (1.36∗10^7^) and D (1.48∗10^9^).

Discovery of lacritin isoforms C and D by MS has important biological implications for contextualizing the multifaceted role of lacritin at the ocular surface. Over 3 decades of literature on lacritin has largely focused on isoform A, leaving the function of other isoforms completely uninvestigated. Most surprisingly, we described the existence of isoform D circulating in tear fluid, an 85 amino acid splice variant without an annotated signal sequence and predicted not to be translated. To better understand the structure of these isoforms, we again performed AlphaFold modeling and showed that both isoform C and D are predicted to adopt an alpha helical structure within their isoform-specific sequences (residues 83–101 and 53–63, respectively). A previous study by Zhang *et al.* (38) on isoform C corroborated an ordered structure around this region, which is unable to bind syndecan-1, leaving its current function unknown. Further analysis of these two helices revealed a difference in length, amino acid identity, and hydrophobicity compared with the C-terminal alpha helix observed in isoform A (Fig. S4). Taken together, this would support the previous observations on isoform C made by Zhang *et al.*, though it remains unclear whether isoform D would be able to engage syndecan-1 without further biochemical characterization. Notably, isoform D contains Gln and Lys acceptor residues for TGM2-mediated crosslinking to form multimers. Given that isoform A and C have previously been shown to form multimers, isoform D may also multimerize.

Lastly, we performed multiple sequence alignment analysis, which revealed that the evolutionary conservation of these isoforms dates back only to a few primates, whereas the densely O-glycosylated region of lacritin seems to remain conserved across a wider range of organisms (Fig. S5). The evolutionary persistence of the mucin domain within lacritin suggests a key biological role associated with this densely O-glycosylated region. It is possible that the mucin domain serves a protective role against proteases (50, 51), acts as a ligand for galectin-3 in tear fluid (52), or mediates lacritin crosslinking, which can be elucidated in future studies.

### Uncovering the tear fluid glycoproteome

**Figure 5:**
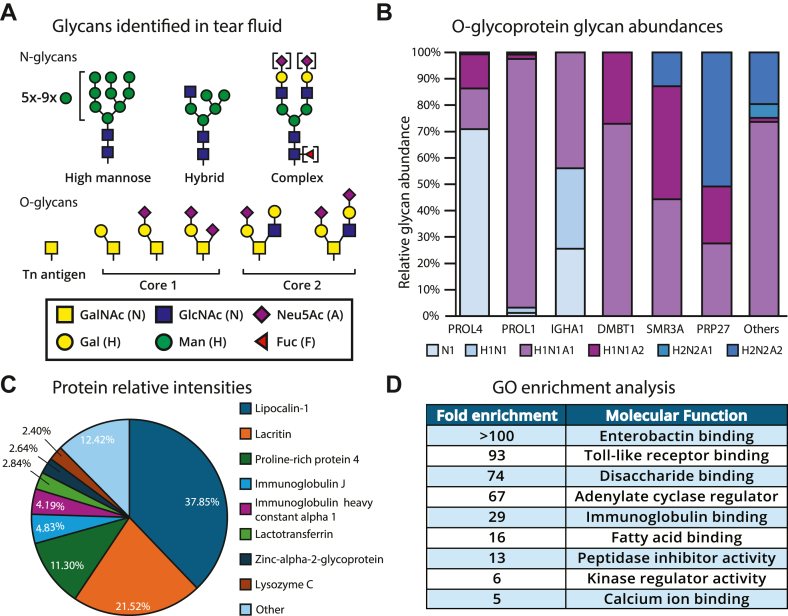
**Uncovering the tear fluid glycoproteome.** (A) N- and O-glycan structures identified in this study using GlycoFASP method. *B*, O-glycosylation landscape of different O-glycoproteins where H represents Hexose, N represents HexNAc, and A represents Neu5Ac. *C*, protein relative abundances from GlycoFASP. *D*, Gene Ontology (GO) enrichment analysis of all glycoproteins identified from both enrichment methods. GlycoFASP, O-glycosylation-focused filter-aided sample preparation.

Beyond lacritin, we performed the most in-depth analysis of the tear fluid glycoproteome to date. Here, we report a range of site-localized N- and O-glycans, including high mannose, hybrid, and complex (sialylated, fucosylated, and sialofucosylated) N-glycans and Tn antigen, core 1, and core 2 O-glycans (Fig. 5*A*). While this corroborates previous N- and O-glycomic studies on tear fluid (22, 23, 39, 40), we note that glycosite specificity and protein identity are inherently lost in glycomics experiments. Therefore, we aimed to elucidate the site-specific glycosylation landscape of O-glycoproteins identified with GlycoFASP, which allowed us to observe vastly diverse O-glycosylation profiles unique to individual proteins (Fig. 5*B* and Table S1). For instance, proline-rich protein 4 (PROL4) primarily displayed the Tn antigen, whereas deleted in malignant brain tumors 1 (DMBT1) was predominantly sialylated. This may suggest that these proteins derive from different ocular cell types with distinct glycosyltransferase expression levels. Alternatively, the sequence of the glycoprotein itself may exhibit different propensities for glycan extension based on solvent accessibility and specific sequence motifs for extending glycosyltransferases (53). To better understand the function of glycoproteins captured by GlycoFASP, we performed relative quantitation (Fig. 5*C*) and Gene Ontology enrichment analysis (Fig. 5*D*). Overall, the top 10 highest intensity proteins identified with this method overlap with previous proteomic analyses of tear fluid, and the biological functions of the broader glycoproteome also support previous observations (24, 54, 55). Lastly, we direct readers to the Glycosites Supplementary Table tab in Table S1 for a detailed list of all identified glycoproteins with localized O-glycosites and corresponding glycan structures as a reference resource.

### In-depth glycosylation characterization of secretory IgA1 and IgJ in tears

**Figure 6:**
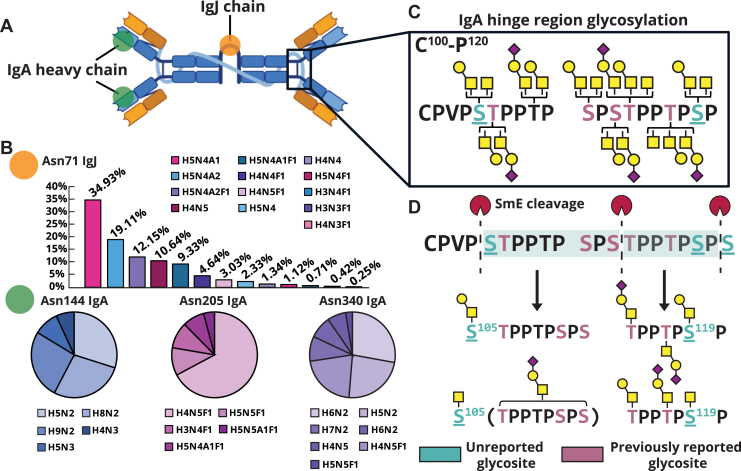
**IgA and IgJ site-specific N-/O-glycan analysis.***A*, *cartoon representation* of IgA dimer held together by IgJ. *B*, site-specific O-glycosylation of IgA hinge region with previously unreported glycosites colored in *teal* and previously reported glycosites colored in *pink*. *C*, site-specific N-glycan analysis of IgA and IgJ where H represents Hexose, N represents HexNAc, F represents Fucose, and A represents Neu5Ac. *D*, representation of glycopeptides generated by mucinase SmE to enable glycosite localization. IgA, immunoglobulin A, IgJ, immunoglobulin J; SmE, *Serratia marcescens* Enhancin.

Immunoglobulin A1 (IgA1) is the most abundantly expressed immunoglobulin in the human body, where it plays an important role in mucosal immunity and protection against pathogens. Structurally, it is often found as a secreted homodimer joined together by an N-glycosylated immunoglobulin J (IgJ) chain (Fig. 6*A*) Here, IgA glycans are known to facilitate its binding to mucosal surfaces, promote pathogen clearance, and confer resistance to proteolysis by bacterial proteases (56, 57). Given that IgA1 has documented glycan alterations in various diseases (58, 59), we asked whether our workflow could enable deep glycoproteomic profiling of IgA1 and its glycoforms, with the goal of informing future diagnostic tools. Notably, previous work revealed that aberrant glycosylation of IgA1 plays a key role in the pathogenesis of autoimmune diseases such as IgA nephropathy and IgA vasculitis (60, 61, 62). Furthermore, altered glycosylation of serum IgA1 is associated with ovarian cancer, breast cancer, colorectal cancer, and hepatitis B virus–related liver cancer (57, 60, 63, 64). While serum IgA has been extensively investigated as a glycan-specific disease biomarker, tear fluid IgA and its specific glycoforms have yet to be elucidated, where its glycosylation could be diagnostically informative for diseases such as DED. Here, we reasoned that if our workflow could capture tear fluid IgA glycoepitopes, then our enrichment strategy could be applied in the future to profile IgA glycosylation in ocular diseases where aberrant glycosylation may be prevalent.

Previous studies reported a total of six O-glycosylation sites in the IgA hinge region (HR), where an increase in the Tn antigen or sialyl Tn antigen (Neu5Acα2–6GalNAcα–Ser/Thr) and a decrease in sialyl T antigen (Galβ1–3GalNAcα1–Ser/Thr) are concomitant with IgA nephropathy (58, 65, 66, 67). Historically, characterization of IgA1 HR glycosylation has been analytically challenging (66, 67). This is evidenced by the fact that previous glycoproteomic studies of IgA1 were performed on highly purified IgA1, necessitating both immunoprecipitation and size-exclusion chromatography procedures on large quantities of human serum (65, 68). In this study, we demonstrated full coverage of the IgA1 HR from just 50 μg of tear fluid, where we site-specifically localized Tn and (sialyl) T antigen O-glycan structures to all six of the previously reported glycosites (shown in *pink*) (Fig. 6, *C* and *D*). In addition to these known glycosites, we identified two previously unreported glycosites (*teal*), which we were able to localize *via* digestion with SmE (Fig. 6*D*). The glycopeptides generated by SmE are depicted with their corresponding O-glycan structures. Overall, we describe comprehensive coverage of the IgA1 HR without tedious purification procedures while requiring minimal sample input. This not only represents the first characterization of IgA1 glycoforms in tear fluid but also highlights the sensitivity of our workflow, which we envision can be applied to other biofluids with ease.

In addition to mapping the densely O-glycosylated HR, we performed site-specific N-glycan quantification of Asn71 on IgJ and Asn144, 205, and 340 on IgA1 (Fig. 6*B*). Interestingly, sialylated and sialofucosylated N-glycan structures were highly expressed on IgJ, whereas IgA1 exhibited almost no sialylation on its N-glycan structures (∼10% relative abundance on only Asn205 and no other glycosites). Further, the N-glycan structures modifying the three N-glycosites of IgA1 varied drastically, as evidenced by the extensive fucosylation on Asn205 when compared with Asn144 (no fucosylation) and Asn340 (<10% relative abundance of fucosylation). Finally, high mannose N-glycans seemed to predominate on Asn144 and Asn340, though complex N-glycans were also detected at these sites. Our results corroborated previous tear fluid N-glycomics studies, which suggested that tear fluid N-glycans are primarily complex structures and heavily fucosylated, which was observed on Asn71 of IgJ and Asn205 of IgA1 (69). In addition, the identified high mannose structures on Asn144 and Asn340 of IgA1 have similarly been reported in a separate N-glycoproteomic study on serum and salivary IgA1 (68).

Beyond IgA1 and IgJ, we also investigated N-glycosylation at Asn497 on lactoferrin (Fig. S6), a highly abundant glycoprotein in tear fluid (making up 25% of total tear protein abundance) (70) with known changes in expression levels in DED. Lactoferrin plays important roles at the ocular surface through its antibacterial activity, anti-inflammatory properties, and ability to promote cell proliferation. While the role of glycans on lactoferrin is not well understood, alterations to its N-glycan structures have been reported in several diseases. As glycosylation changes to IgA1 and lactoferrin have yet to be established for ocular pathologies, we are optimistic that future application of our workflow could unravel potential disease-specific glycosylation changes.

## Conclusion

This study represents the most in-depth analysis of the site-specific glycosylation landscape of tear fluid to date. To overcome previous analytical challenges in studying tear fluid, we harnessed recently introduced mucinases and unique enrichment techniques, which enabled unprecedented coverage of the tear fluid glycoproteome. We highlight the versatility of our methods through site-specific profiling of glycosylated proteins, which are established diagnostic biomarkers and key regulators of ocular homeostasis, namely, IgA1 and lacritin. As we only investigated a single patient for this study, we envision expanding our workflow to larger patient cohorts in the future with the goal of elucidating site-specific glycoepitopes for ocular disease diagnosis.

Crucially, lacritin has been extensively studied over the past 30 years and is the only therapeutic for DED currently in clinical trials. Despite this, the glycans that modify lacritin, which comprise more than half its molecular weight, remained a critical blind spot until now. Leveraging our site-specific glycomap of lacritin, we employed two protein modeling programs, GlycoShape and AlphaFold 3.0, to better understand the structure of lacritin. Ultimately, we provided new insight into the complex biology mediated by lacritin, corroborating previous observations by the Laurie group, while raising new possibilities for the role of specific lacritin glycoforms and isoforms.

## Experimental procedures

### Human tear fluid sample collection

The collection of tear fluid from a healthy patient from a biobank and subsequent analysis was approved by Helse Sør-Øst Regional Committee for medical and health research in Norway (application no.: 427997). Healthy (asymptomatic) individuals in the biobank were defined as having a Dry Eye Questionnaire 5 score of <7 and an Ocular Surface Disease Index of <12, in addition to having a healthy fluorescein Tear film Break-Up Time >9 s. Individuals with previous history of DED are excluded from biobank of controls. The analysis was performed on a single asymptomatic individual with no sign of DED. Ethical consideration and privacy precluded disclosing detailed information about the individual in publications, only to be provided on a group basis. Participant had given informed consent for the study. Tear fluid from this single volunteer was collected on Schirmer tear strips (placed onto the lower eyelid for 5 min) or by microcapillary tubes. We confirm that the human studies reported here abide by the Declaration of Helsinki principles.

## MS sample preparation

### GlycoFASP of tear fluid

Tear fluid proteins were directly extracted and reduced from tear strips by using either 0.5% sodium deoxycholate in 5 mM DTT and 20 mM Tris (65 °C 1 h) or 4 M urea in 5 mM DTT and 20 mM Tris (23 °C, 4 h). Next, proteins were alkylated with 10 mM IAA (15 min in the dark at 23 °C) and subsequently washed six times with 20 mM Tris on a 50 kDa molecular weight cutoff filter. On-filter digestion was performed with mucinase SmE (enzyme:substrate ratio of 1:20), which was allowed to react overnight at 37 °C. Glycopeptides generated from mucinase digestion can now pass through the 50 kDa filter and are collected for subsequent tryptic digestion at a 1:50 enzyme:substrate ratio for 6 h at 37 °C. All reactions were quenched by adding 1 μl of formic acid and diluted to a volume of 200 μl prior to desalting. Desalting was performed using 10 mg Strata-X 33 μm polymeric reversed phase SPE columns (Phenomenex). Each column was activated using 500 μl of acetonitrile (ACN) (Honeywell) followed by of 500 μl of 0.1% formic acid, 500 μl of 0.1% formic acid in 40% ACN, and equilibration with two additions of 500 μl of 0.1% formic acid. After equilibration, the samples were added to the column and rinsed twice with 200 μl of 0.1% formic acid. The columns were transferred to a 1.5 ml tube for elution by two additions of 150 μl of 0.1% formic acid in 40% ACN. The eluent was then dried using a vacuum concentrator (LabConco) prior to reconstitution in 10 μl of 0.1% formic acid. The resultant peptides were then injected onto a Dionex Ultimate3000 coupled to a Thermo Orbitrap Eclipse Tribrid mass spectrometer. We employed a higher-energy collision dissociation (HCD) product-dependent electron transfer dissociation (ETD) method; in some cases, we used supplemental activation in ETD (EThcD). The files were searched using Byonic, followed by manual data curation.

### Preparation of StcE^E447D^ beads

The pET28 plasmids for His-tagged SmEnhancin and StcE were kindly provided by the Bertozzi laboratory. StcE^E447D^ was conjugated to *N*-hydroxysuccinimide (NHS)-activated beads and washed three times with 1 ml of PBS followed by the addition of the proteins, which were allowed to bind overnight at 4 °C. Free NHS-esters were capped by adding 100 mM Tris, pH 7.4, to the bead slurry for 20 min at 4 °C. Bicinchoninic acid assays were performed after the reactions in order to determine the sufficient binding efficiency. After capping, beads were washed three times with a high-salt buffer (20 mM Tris and 500 mM NaCl) followed by a buffer without salt (20 mM Tris).

### StcE^E447D^ enrichment of tear fluid

Alternatively, tears collected from capillary tubes (diluted to 500 μl with PBS with a final concentration of 0.5 mg/ml) were loaded onto StcE^E447D^-conjugated NHS beads, washed three times with 20 mM Tris and 500 mM NaCl, and eluted with 0.5% sodium deoxycholate. Following elution, the mucin-enriched tear fluid was subjected to reduction, alkylation, and proteolytic digestion as previously described. All reactions were quenched by adding 1 μl of formic acid and diluted to a volume of 200 μl prior to desalting. Addition of formic acid also caused sodium deoxycholate to precipitate out of the solution, and the resulting supernatant was transferred to a new tube before desalting. Desalting and sample injection into the mass spectrometer was performed as previously described in the GlycoFASP methods section.

### Unenriched tear fluid processing

Proteins extracted from tear fluid were diluted to a final concentration of 0.2 mg/ml in 100 μl of PBS. DTT was then added to a concentration of 2 mM and reacted at 65 °C for 1 h followed by alkylation in 5 mM IAA for 15 min in the dark at room temperature. Subsequently, digestion with trypsin was done at a 1:50 enzyme:substrate ratio for 6 h at 37 °C. All reactions were quenched by adding 1 μl of formic acid and diluted to a volume of 200 μl prior to desalting. Desalting and sample injection into the mass spectrometer was performed as previously described in the GlycoFASP methods section.

### MS data acquisition

Samples were analyzed by online nanoflow liquid chromatography–tandem MS using an Orbitrap Eclipse Tribrid mass spectrometer (Thermo Fisher Scientific) coupled to a Dionex UltiMate 3000 HPLC (Thermo Fisher Scientific). For each analysis, 4 μl was injected onto an Acclaim PepMap 100 column packed with 2 cm of 5 μm C18 material (Thermo Fisher; catalog no.: 164564) using 0.1% formic acid in water (solvent A). Peptides were then separated on a 15 cm PepMap RSLC EASY-Spray C18 column packed with 2 μm C18 material (Thermo Fisher; catalog no.: ES904) using a gradient from 0 to 35% solvent B (0.1% formic acid with 80% acetonitrile) in 60 min. Full scan MS1 spectra were collected at a resolution of 60,000, an automatic gain control target of 3e5, and a mass range from *m/z* 300 to 1500. Dynamic exclusion was enabled with a repeat count of 2, repeat duration of 7 s, and exclusion duration of 7 s. Only charge states 2 to 6 were selected for fragmentation. MS2s were generated at top speed for 3 s. HCD was performed on all selected precursor masses with the following parameters: isolation window of 2 *m/z*, 29% normalized collision energy, orbitrap detection (resolution of 7500), maximum injection time of 50 ms, and a standard automatic gain control target. An additional ETD fragmentation of the same precursor was triggered if (1) the precursor mass was between *m/z* 300–1500 and (2) three of eight HexNAc or NeuAc fingerprint ions (126.055, 138.055, 144.07, 168.065, 186.076, 204.086, 274.092, and 292.103) were present at *m/z* ±0.1 and greater than 5% relative intensity. Two files were collected for each sample: the first method collected an ETD scan with supplemental energy (EThcD), whereas the second method collected a scan without supplemental energy. Both used charge-calibrated ETD reaction times, 100 ms maximum injection time, and standard injection targets. EThcD parameters were as follows: Orbitrap detection (resolution 7500), calibrated charge-dependent ETD times, 15% normalized collision energy for HCD, maximum injection time of 150 ms, and a standard precursor injection target. For the second file, dependent scans were only triggered for precursors below *m/z* 1000, and data were collected in the ion trap using a normal scan rate.

### MS data analysis

Raw files were searched using Byonic (version 4.5.2; Protein Metrics, Inc) against the UniProtKB/Swiss-Prot *Homo sapiens* proteome (query: proteome:up000005640 AND reviewed:true) and a curated mucin database, which was generated from a previous study. Briefly, the mucin database consists of nearly 350 proteins from UniProt’s annotated human proteome predicted to bear the dense O-glycosylation characteristic of mucin domains. For all samples, we used the default O-glycan database containing nine common structures. Raw files were first searched against the human proteome and then the curated mucin database. In both cases, files were searched with semispecific cleavage N-terminal to Ser and Thr and six allowed missed cleavages. Samples treated with trypsin were not only searched with the same parameters but also allowed cleavage C-terminal to Arg or Lys. Mass tolerance was set to 10 ppm for MS1s and 20 ppm for MS2s. Met oxidation was set as a variable modification, and carbamidomethyl Cys was set as a fixed modification. From the Byonic search results, glycopeptides were filtered to a score of >200 and a logprob of >2. From the remaining list of glycopeptides, the XICs, full mass spectra (MS1s), and fragmentation spectra (MS2s) were investigated in XCalibur QualBrowser (Thermo) to generate a list of true-positive glycopeptides, as reported in Table S1. Each reported glycopeptide listed in Table S1 was manually validated from the filtered list of Byonic’s reported peptides (score >200 and logprob >2) according to the following steps: The MS1 was first used to confirm the precursor mass, and chosen isotope was correct. This also allowed us to identify any coisolated species that could interfere with the MS2s and/or explain unassigned peaks. The HCD and EThcD fragmentation spectra were then investigated to identify sufficient coverage to make a sequence assignment. When possible, multiple MS2 scans were averaged to obtain a stronger spectrum. For HCD, an initial glycopeptide ID was confirmed if the presence of the precursor mass without a glycan present (*i.e.*, Y0), along with coverage of b and y ions without glycosylation. For longer peptides, we required the presence of Y0 and fragments that were expected to be abundant (*e.g.*, N-terminally to Pro, C-terminally to Asp). When the peptide contained a Pro at the C terminus, the b_n-1_ was considered sufficient. Further, when the sequence contained oxidized Met, the Met loss from the bare mass was considered as representative of the naked peptide mass. We then used electron-based fragmentation MS2 spectra for localization. Here, all plausible localizations were considered, regardless of search result output. We confirmed the presence of fragment ions in ETD or EThcD that were between potential glycosylation sites; if sufficient c/z ions were present, then a glycan mass was considered localized. For glycopeptide manual validation, XICs are evaluated at the MS1 level to determine the charge and *m/z* of the highest abundance precursor species. MS data files and raw search output can be found on PRIDE with identifier PXD064788.

### Lacritin protein modeling with AlphaFold 3.0 and GlycoShape

The most abundant O-glycan structures of lacritin were first calculated at each O-glycosite using LFQ of AUC intensities of XICs of all lacritin glycopeptides identified (Data S1). Once glycan structures were determined, lacritin’s UniProt ID (Q9GZZ8) was fetched on GlycoShape webserver’s Re-Glyco tab (https://glycoshape.org/reglyco). Corresponding glycans were then manually input using the “Advanced (Site-by-Site) Glycosylation” feature before hitting “process” for a predicted structure. AlphaFold 3.0 was accessed through the AlphaFoldServer website, and the canonical sequence (UniProt ID: Q9GZZ8) of lacritin was input manually. Next, glycan structures were selected using 3-letter Chemical Component Dictionary codes. Here, NAG was used for a single HexNAc residue, BGC was used for a beta-linked hexose residue, and FUC was used for a fucose residue. The resultant Protein Data Bank file with the predicted fold was input into CHARMM-GUI for further glycan editing and protein solvation before being loaded onto PyMOL 2.0 for glycan recoloration and better visualization. For lacritin C-terminal alpha helix modeling, the GlycoShape Protein Data Bank file was exported to PyMOL 2.0, and only the C-terminal helix (amino acids 108–138) was shown with the rest of the protein sequence marked as hidden. For isoform C-terminal structure analysis, AlphaFold 3.0 was used as described previously.

## Data availability

All MS data and search results acquired for this article have been deposited on the PRIDE repository. Reviewers can access raw data with the following login information: Project accession: PXD064788; Token: fO49eEArej9P. reviewer_pxd064788@ebi.ac.uk.

## Supporting information

This article contains supporting information.

## Conflict of interest

S. A. M. is a coinventor on a Stanford patent related to the use of mucinases as research tools. All other authors declare that they have no conflicts of interest with the contents of this article.
